# Mechanical behavior of cemented paste backfill under the coupled effect of loading rate and moisture content

**DOI:** 10.1371/journal.pone.0334084

**Published:** 2026-05-15

**Authors:** Chuanchuan Wan, Xiaocong Yang, Jiwei Bian, Wenyuan Xu, Hongwei Shi, Hao Huang

**Affiliations:** 1 School of Civil and Resource Engineering, University of Science and Technology Beijing, Beijing, China; 2 BGRIMM Technology Group, Beijing, China; 3 Technology Engineering Subsidiary of Yunnan Chihong Zinc&Germanium Co., Ltd，Beijing, China; Graphic Era Deemed to be University, INDIA

## Abstract

This study investigates the coupled effects of loading rate and moisture condition on the mechanical behavior of cemented paste backfill (CPB). Uniaxial compression tests were conducted on CPB specimens under dry (0%), nature (18%), and saturated (32%) moisture conditions at loading rates ranging from 0.05 to 2.0 mm/min. The results show that uniaxial compressive strength (UCS) follows a second-order polynomial relation with loading rate and exhibits an overall rise-then-fall trend, with a common transition region around 1.0 mm/min. Relative to the nature condition, the dry condition generally enhances strength and stiffness, whereas the saturated condition weakens them but increases peak strain over the low-to-intermediate loading-rate range. Failure mode is also moisture dependent, with dry specimens showing more brittle behavior and saturated specimens exhibiting more tensile-dominated failure. The nonlinear index is likewise sensitive to loading rate and moisture condition, and saturated CPB exhibits the highest nonlinear index before the transition region. Based on UCPI analysis, the fitted optimal loading rates are 1.20, 1.23, and 0.97 mm/min for the dry, nature, and saturated conditions, respectively. Although the fitted optima differ slightly, they remain clustered around 1.0 mm/min. These findings indicate that loading-rate regulation in underground backfill-supported environments should account for the prevailing moisture condition rather than rely on a single fixed loading-rate criterion.

## 1. Introduction

Backfill mining has emerged as a primary method for deep mineral extraction due to its advantages in safety, efficiency, cost-effectiveness, and environmental sustainability [1,2]. It not only improves ore recovery and reduces production costs, but also mitigates geological hazards such as surface subsidence and groundwater decline, while protecting the ecological environment. As a permanent structural material that supports the roof and overburden, cemented paste backfill (CPB) plays a crucial role in ensuring mine stability and sustainable development.

The long-term stability of CPB is critical for supporting the roof, overburden, and controlling ground movement in underground mines [3]. Previous research has identified key factors influencing CPB solidification and long-term strength, including temperature, solid mass concentration, and the sand-to-cement ratio. Specifically, temperature affects hydration reactions during curing [4]. solid mass concentration governs the flowability and strength of the slurry [5,6], and the sand-to-cement ratio strongly influences the setting and mechanical performance of the backfill [7,8]. These findings provide valuable guidance for ensuring safe and reliable mine production.

As shallow mineral resources are depleted, mining has shifted toward deeper levels, where complex conditions such as high in-situ stress, elevated ground temperature, high permeability, and strong mining induced disturbances have become increasingly critical [9–11]. Among these, stress concentration caused by high in-situ stress and the dynamic effects of rapid loading are particularly critical [12–14]. Previous studies have demonstrated that loading rate strongly influences the mechanical behavior of rocks and rock-like materials: while a moderate increase in loading rate can enhance CPB strength, this effect reaches a critical loading rate, beyond which strength rapidly deteriorates. Correspondingly, the failure mode evolves from mixed tensile–shear at low rates to shear- or tensile-dominated failure at high rates [15]. By applying damage mechanics theory and advanced micro-scale monitoring techniques such as acoustic emission (AE), computed tomography (CT), scanning electron microscopy (SEM), and digital image correlation (DIC), researchers have revealed the mechanisms that restrict crack propagation at high loading rates [16,17]. Additionally, numerical simulations and monitoring-based approaches have been employed to establish constitutive damage models and 3D simulations of CPB and rock, using loading rate as a critical parameter to capture the evolution of strength and failure processes [9,18,19]. Theoretical studies further indicate a polynomial or exponential relation be-tween loading rate and CPB compressive strength [16,20], providing a theoretical basis for design optimization and failure prediction. However, most existing studies treat loading rate as an isolated factor, with limited attention given to its interactions with other variables, such as moisture content, in deep mining conditions.

Groundwater is another key factor in deep mining, where CPB remains underground for long periods and inevitably undergoes moisture variation [21]. Water significantly influences the mechanical behavior of CPB [22,23]. Previous studies indicate that as moisture content increases, CPB strength decreases, with the dry state showing the highest com-pressive strength [24–26]. At the same time, higher moisture content enhances ductility, slows crack propagation, and produces a more gradual failure process. However, excessive water raises pore-water pressure, weakens particle bonding, and accelerates crack initiation and propagation, ultimately reducing resistance to damage [27–29]. Under actual deep-mining conditions, the mechanical response of CPB is governed by the combined effects of both loading rate and moisture content. Although each factor has been studied separately, systematic investigations of their coupled effects are still limited. Therefore, clarifying the mechanical behavior of CPB under the combined action of loading rate and moisture is crucial for reproducing field conditions and bridging this research gap.

In this study, uniaxial compression tests were carried out on CPB specimens conditioned under three moisture conditions (dry 0%, nature 18%, and saturated 32%) and six loading rates to investigate their coupled effects on strength, elastic modulus, stress-strain behavior, and failure mode. Based on the test results, rate-selection criteria and operating bounds tailored to different moisture conditions are proposed. A nonlinear index is introduced to characterize ductility and the nonlinear mechanical response of CPB. By treating loading rate and moisture content as co-variables, this study clarifies their combined influence under deep-mining conditions and provides both theoretical insights and practical recommendations for backfill design and mine safety.

## 2. Materials and methods

### 2.1. Materials

#### 2.1.1. Manufactured sand.

**Fig 1:**
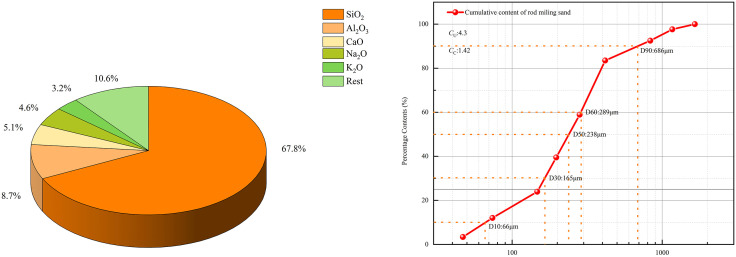
Composition and particle size distribution of manufactured sand. (a) the composition and proportions of manufactured sand. (b) the particle size distribution curve of manufactured sand.

The manufactured sand used in this experiment is sourced from a nickel mine in Gansu. The chemical properties of the manufactured sand were tested using an X-ray fluorescence spectrometer. As shown in Fig 1a, the manufactured sand is primarily composed of SiO₂, Al₂O₃, and CaO, accounting for more than 81.6 wt.%, which is beneficial for the development of the compressive strength of the CPB. Fig 1b shows the manufactured sand’s particle size distribution. The uniformity coefficient (*C*_*U*_) and curvature coefficient (*C*_*C*_) of the sand particles, calculated as (d60/d10) and (d30^2^/d60 × d10), are 4.3 and 1.42, respectively, indicating that the particles of the sand are relatively uniform, with a complete and continuous particle size distribution.

#### 2.1.2. Cement and water.

**Fig 2:**
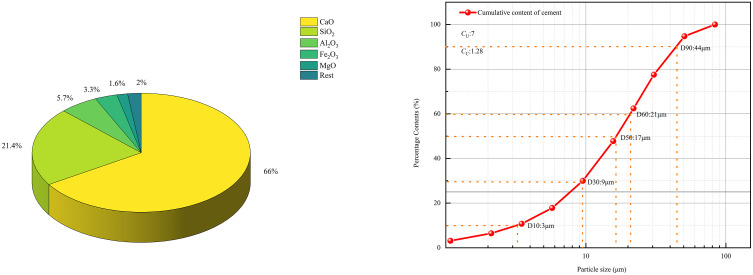
Composition and particle size distribution of cement. (a) the composition and proportions of cement. (b) the particle size distribution curve of cement.

The cementing material used in this experiment is the commonly used Portland cement (P.042.5) in the mining industry. The 28-day UCS (unconfined compressive strength) is 42.5 MPa, and the bending strength is 6.5 MPa. The cement’s specific gravity and surface area are 3.2 and 1.13 m²/g, respectively. The chemical composition and particle size distribution of the Portland cement are shown in Fig 2. The uniformity coefficient (*C*_*U*_) and curvature coefficient (*C*_*C*_) of the Portland cement particles, calculated as (d60/d10) and (d30^2^/d60 × d10), are 7 and 1.28, respectively, indicating that the cement particles have a complete range of particle sizes and relatively regular shapes. The mixing water used was tap water from the laboratory.

### 2.2. Experimental Variables

The value ranges and grouping of the two experimental variables, loading rate and moisture content, were determined through theoretical analysis, literature review, and field sampling from underground mining sites.

#### 2.2.1. Loading rate variable selection.

**Fig 3:**
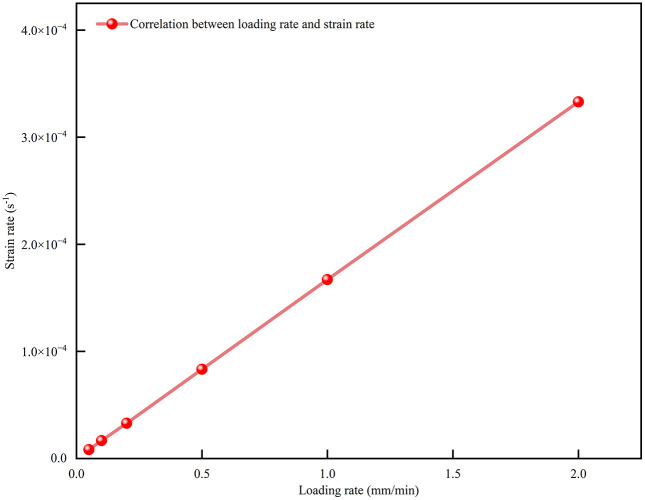
Correlation between loading rate and strain rate.

In practical mining operations, CPB is subjected to varying deformation rates of surrounding rock loads due to differences in mining depth, extraction methods, and disturbance intensity. According to previous studies, the loading rate associated with the interaction between surrounding rock and CPB in underground mines typically falls within a range of 0.05 to 2.0 mm/min [13]. This range corresponds to low-rate conditions, such as gradual mining or long-term creep, and high-rate conditions, such as rapid extraction or sudden dynamic disturbances. The corresponding strain rates for these loading rates are depicted in Fig 3.

Therefore, this study selected six representative loading rates (0.05 mm/min, 0.1 mm/min, 0.2 mm/min, 0.5 mm/min, 1.0 mm/min, and 2.0 mm/min) to reflect typical in-situ mining scenarios. These loading rates were used to systematically investigate the effects of loading rate on the strength, deformation characteristics, and failure modes of CPB.

#### 2.2.2. Moisture content variable selection.

In underground mining environments, CPB is subject to variable moisture conditions due to groundwater seepage, evaporation, and mine ventilation. These conditions are typically categorized as dry (areas with minimal groundwater or strong ventilation), nature (ambient humidity equilibrium), and saturated (areas with persistent or high-water exposure). As is shown in Table 1, field sampling defined the corresponding moisture content ranges: dry (1–3%), nature (16–20%), and saturated (30–34%). Three representative moisture contents (0%, 18%, and 32%) were selected to simulate these field conditions. This provides a realistic basis for evaluating the coupled effects of moisture condition and loading rate on the mechanical behavior of CPB.

**Table 1:** Field Sampling Moisture Content Test Results.

Sampling Environment	Initial Mass (g)	Dry Mass (g)	Moisture Content (%)
Dry Environment	294.3	291.5	0.96
	306.2	300.7	1.83
	319.1	311.1	2.57
Nature Environment	267.4	230.1	16.2
	291.5	248.3	17.4
	304.6	254.9	19.5
Saturated Environment	349.2	267.8	30.4
	323.7	247	31.1
	309.8	231.4	33.9

### 2.3. Experimental methods

**Fig 4:**
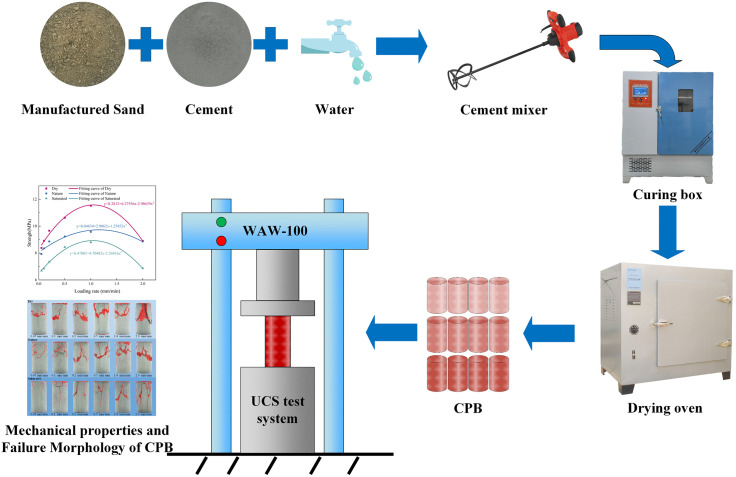
The experimental procedure.

Fig 4 shows the experimental procedure, which mainly includes preparing CPB samples, drying, water saturation, and uniaxial compressive strength testing.

#### 2.3.1. Preparation of CPB Samples.

In this experiment, the sand-to-cement ratio (the dry mass ratio of cement to manufactured sand) and the mass concentration were set to 1:4 and 75%, respectively. The preparation process for CPB samples is as follows:

(1) Weigh an appropriate amount of manufactured sand and cement dry material and mix them evenly;
(2) Add the required amount of water and stir the mixture in a mechanical mixer for 5 minutes until the slurry is evenly mixed;
(3) Pour the evenly mixed filling slurry into a cylindrical mold (inner diameter 50 mm, inner height 100 mm), and leave it to stand for 24 hours to set. After demolding, the samples are placed in a constant temperature and humidity curing chamber (temperature of (20 ± 1) ℃, humidity 95%) for curing. After 28 days, the samples are removed for subsequent tests.

#### 2.3.2. Saturation and drying treatment of CPB samples.

The CPB samples, cured for 28 days, were subjected to vacuum saturation for 6 hours using a vacuum saturation apparatus. After removal, the surface of the CPB samples was wiped dry. The saturated samples were wrapped in cling film to prevent moisture evaporation from affecting the test results. The CPB samples were then dried in an oven at 60°C for 24 hours until a constant weight was reached. To compare the effects of saturation and drying on the CPB, neither saturated nor dried samples were defined as nature CPB. In addition, the CPB samples in the dry, nature, and saturated states were weighed using an electronic scale with an accuracy of 0.001g, and the moisture contents under each condition were determined to be 0%, 18%, and 32%, respectively.

#### 2.3.3. Unconfined compressive strength testing.

By the ASTM C39/C39M-16 standard, unconfined compressive strength (UCS) tests were conducted on the treated CPB samples using an electro-hydraulic servo universal testing machine (WAW-100, Changchun, China). The axial compression was displacement-controlled, with loading rates set at 0.05 mm/min, 0.1 mm/min, 0.2 mm/min, 0.5 mm/min, 1.0 mm/min, and 2.0 mm/min. During the loading process, the test system automatically recorded the load, displacement, and time data, ultimately obtaining the stress-strain curves of CPB samples under different loading rates and states. Each group of samples was tested three times, and the average value was calculated as the final UCS for each sample type.

#### 2.3.4. Scanning electron microscopy analysis.

After the uniaxial compression tests, representative failed specimens under the dry, nature, and saturated conditions at loading rates of 0.5 mm/min and 2.0 mm/min were selected for scanning electron microscopy (SEM) observation. The specimens were dried at low temperature to a constant mass, and small samples with dimensions no more than 10 mm × 10 mm × 10 mm were extracted from the fracture surfaces. The samples were affixed onto specimen mounts using conductive adhesive and sputter-coated with gold to improve conductivity. High-magnification SEM observations were then carried out on the fracture surfaces to analyze the particle–matrix interfacial bonding state, local debonding features, microcrack distribution, and pore-structure differences under different conditions.

## 3. Results

### 3.1. Effect of loading rate and moisture content on the UCS of CPB

**Fig 5:**
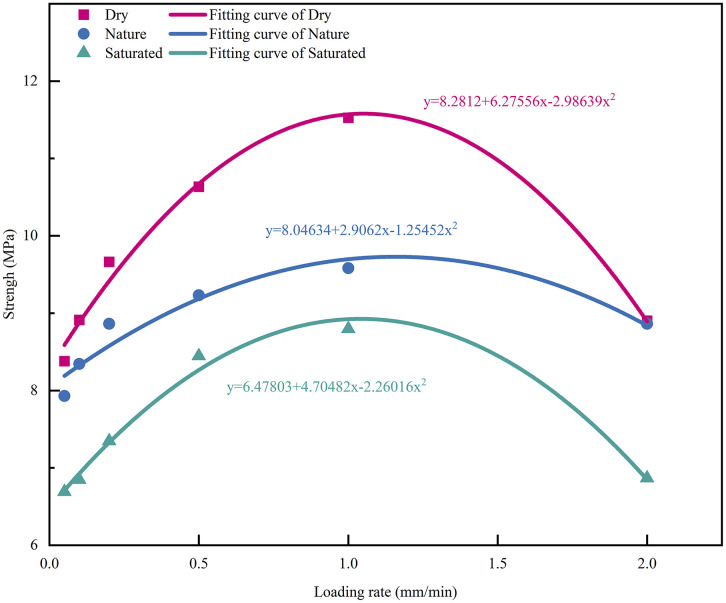
UCS of CPB at different loading rates and moisture contents.

Fig 5 summarizes the UCS of CPB under three moisture conditions (dry, nature, and saturated) at loading rates of 0.05–2.0 mm/min. For each moisture condition, UCS first increases and then decreases with increasing loading rate, and this rise then fall trend fits a second order polynomial relation with loading rate (R² > 0.90 for all fits). Specifically, UCS in the dry, nature, and saturated states increases from 8.38, 7.39, and 6.69 MPa at 0.05 mm/min to 11.53, 9.58, and 8.80 MPa at 1.0 mm/min (increases of 37.5%, 20.8%, and 31.5%, respectively). When the loading rate further increases to 2.0 mm/min, UCS decreases to 8.90, 8.86, and 6.87 MPa, corresponding to reductions of 22.8%, 7.5%, and 21.9% relative to 1.0 mm/min.

**Fig 6:**
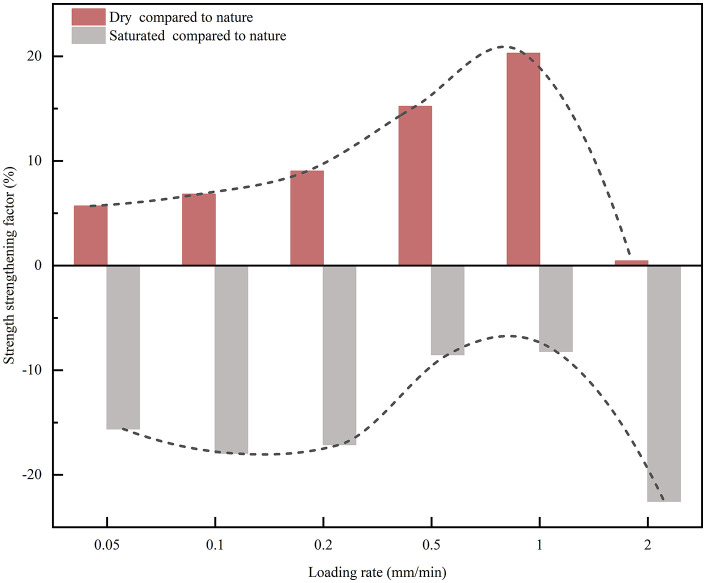
Strength enhancement and deterioration of CPB in dry and saturated states.

At a given loading rate, moisture condition constrains the development of UCS. To quantify moisture induced strengthening (drying) and deterioration (saturation), the nature condition was used as the reference, and the UCS change ratios were computed in Fig 6. The strengthening and deterioration coefficient calculated as follows:


$$\begin{array}{c} \eta_{dry}=\frac{UCS_{dry}-UCS_{nat}}{UCS_{nat}}\times 100\% \end{array}$$ (1)

$$\begin{array}{c} \eta_{sat}=\frac{UCS_{nat}-UCS_{sat}}{UCS_{nat}}\times 100\% \end{array}$$ (2)

Where *UCS*_dry_ is the uniaxial compressive strength of CPB in the dry state; *UCS*_nat_ is the uniaxial compressive strength of CPB in the nature state; *UCS*_sat_ is the uniaxial compressive strength of CPB in the saturated state (MPa).

With increasing loading rate, the strengthening ratio in the dry state increases and reaches a maximum of 20.27% at 1.0 mm/min, and then drops rapidly to nearly zero at 2.0 mm/min. In contrast, the deterioration ratio in the saturated state decreases to a minimum of 8.20% at 1.0 mm/min, but increases sharply to a maximum deterioration of 22.53% at 2.0 mm/min. These opposite trends indicate that loading rate and moisture condition jointly regulate UCS evolution.

The trend of UCS variation with loading rate at different moisture contents suggests that there is a certain critical point for the effect of loading rate on CPB. In this experiment, UCS of CPB shows a noticeable trend change around 1.0 mm/min.

### 3.2. Effect of loading rate and moisture content on the stress-strain behavior of CPB

**Fig 7:**
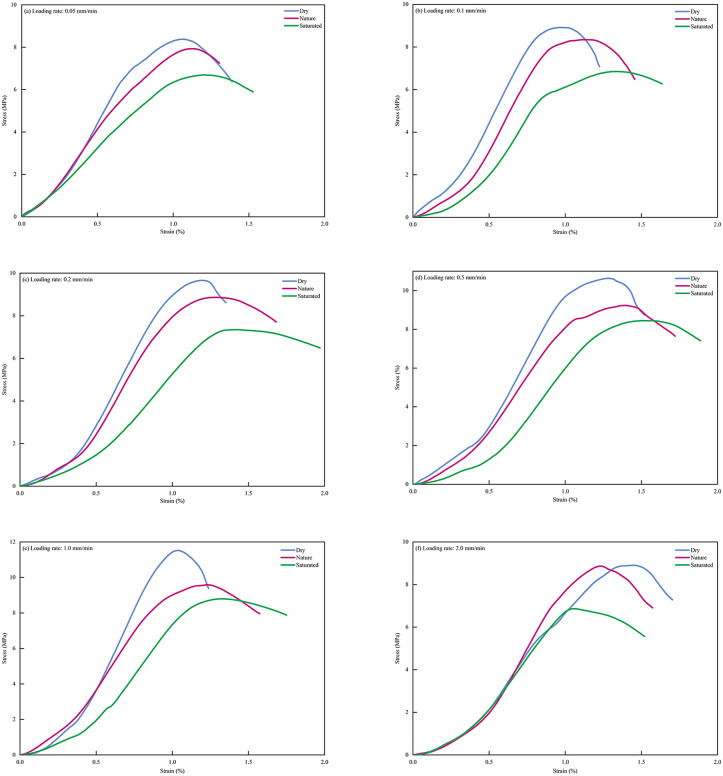
Stress-strain curves of CPB under different conditions. (a) Loading rate:0.05 mm/min. (b) Loading rate:0.1 mm/min. (c) Loading rate:0.2 mm/min. (d) Loading rate:0.5 mm/min. (e) Loading rate:1.0 mm/min. (f) Loading rate:2.0 mm/min.

Fig 7 shows the typical stress-strain curves obtained from uniaxial compression tests on CPB samples with different moisture contents under various loading rates. The stress–strain behavior is generally similar and includes four stages: initial compaction, OA; elastic deformation, AB; plastic yielding, BC; and damage failure, CD. However, significant differences arise due to variations in loading rate and moisture content. Table 2 provides the statistical values of mechanical parameters under different moisture contents and loading rates. Two-way ANOVA showed that both loading rate and moisture condition significantly affected elastic modulus, peak strain, and UCS (*p* < 0.001).The interaction between loading rate and moisture condition was significant for elastic modulus, peak strain, and UCS (both *p* < 0.001) Table 3.

**Table 2:** Statistical Values of Mechanical Parameters Under Different Moisture Contents and Loading Rates (mean ± SD, n = 3).

Loading rate（mm/min）	Moisture content（%）	Elastic Modulus (MPa)	Peak Strain（%）	UCS（MPa）
0.05	0%	12.98 ± 0.68	1.06 ± 0.08	8.38 ± 0.43
	18%	10.12 ± 0.58	1.12 ± 0.07	7.93 ± 0.48
	32%	7.15 ± 0.67	1.21 ± 0.09	6.69 ± 0.45
0.1	0%	14.51 ± 0.66	0.97 ± 0.07	8.91 ± 0.60
	18%	13.38 ± 0.58	1.14 ± 0.08	8.34 ± 0.44
	32%	10.81 ± 0.70	1.33 ± 0.11	6.85 ± 0.59
0.2	0%	13.48 ± 0.51	1.20 ± 0.11	9.66 ± 0.65
	18%	12.44 ± 0.65	1.28 ± 0.10	8.86 ± 0.66
	32%	8.43 ± 0.63	1.43 ± 0.10	7.35 ± 0.56
0.5	0%	14.58 ± 0.70	1.28 ± 0.11	10.63 ± 0.81
	18%	11.71 ± 0.76	1.39 ± 0.11	9.23 ± 0.67
	32%	10.33 ± 0.67	1.52 ± 0.12	8.45 ± 0.61
1.0	0%	18.60 ± 1.04	1.03 ± 0.09	11.53 ± 0.87
	18%	13.06 ± 0.78	1.24 ± 0.09	9.58 ± 0.59
	32%	12.09 ± 0.70	1.32 ± 0.10	8.80 ± 0.58
2.0	0%	10.82 ± 0.94	1.44 ± 0.12	8.90 ± 0.68
	18%	13.72 ± 0.74	1.23 ± 0.09	8.86 ± 0.74
	32%	10.34 ± 0.61	1.05 ± 0.09	6.87 ± 0.63

Initial Compaction Stage (OA): In the initial compaction stage, the initial microcracks, micropores, and defects within the CPB close under the external load, resulting in a concave curve in the stress-strain diagram. Significant differences are observed in this stage due to variations in moisture content. As moisture content increases, porosity and flowability improve, causing an evident extension of the compaction process. For different loading rates, the interaction between moisture content and loading rate is apparent: at lower loading rates, moisture has a more noticeable impact, resulting in longer compaction times; whereas at higher loading rates, the differences become smaller and may even overlap, indicating that both moisture content and loading rate influence this stage;

**Table 3:** Two-way ANOVA results for mechanical parameters.

Response variable	Factor	df	F	*p*
UCS	Loading rate	5	18.177	5.64E-09
	Moisture condition	2	55.697	9.57E-12
	Loading rate × Moisture content	10	10.062	4.15E-04
Elastic modulus	Loading rate	5	41.134	6.60E-14
	Moisture condition	2	167.148	6.02E-19
	Loading rate × Moisture content	10	13.679	1.66E-09
Peak strain	Loading rate	5	9.949	4.88E-06
	Moisture condition	2	10.472	2.60E-04
	Loading rate × Moisture content	10	6.126	2.22E-05

**Fig 8:**
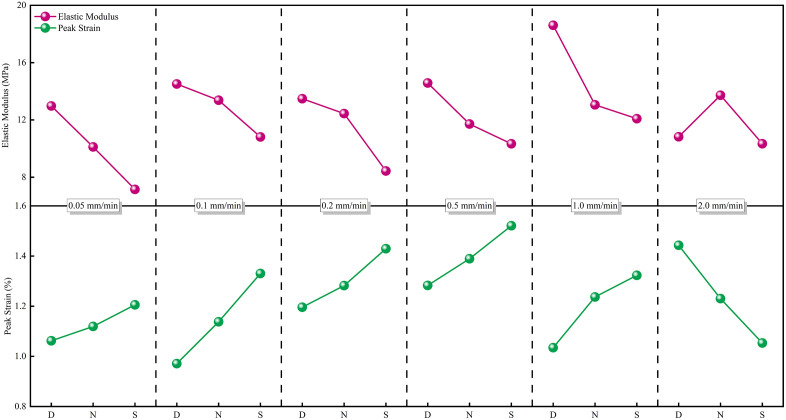
The elastic modulus and the peak strains of CPB under different conditions.

Elastic Deformation Stage (AB): In the elastic deformation stage, the stress-strain relationship of CPB approximates a straight line, and the elastic modulus reflects the material’s resistance to deformation. The elastic modulus is determined by calculating the slope of the stress-strain curve, which is the ratio of stress change to strain change. As shown in Fig 7, as moisture content increases, the elastic modulus of CPB decreases significantly. Specifically, in the dry state, CPB exhibits a higher elastic modulus, indicating that drying enhances its ability to resist deformation; whereas, in the saturated state, excessive moisture significantly weakens CPB’s stiffness. The effect of loading rate on the elastic modulus is more complex. At lower loading rates, the influence of higher moisture content is more pronounced, resulting in a lower elastic modulus. At higher loading rates, the impact of loading rate dominates, causing the influence of moisture content on the elastic modulus to become less significant;

Plastic Yielding Stage (BC): As the loading continues, CPB enters the plastic yielding stage, where cracks gradually expand and propagate, causing the stress-strain curve to become concave downward and approach peak strength. As shown in Fig 7, the stress-strain curve in the plastic yielding stage exhibits significant differences under varying moisture contents and loading rates. Fig 8 demonstrates how moisture content and loading rate affect the peak strain [30]. In this stage, both moisture content and loading rate play significant roles. The peak strain reflects the plasticity or ductility of CPB. Before the critical loading rate, as the moisture content increases, the peak strain follows the order of saturated > nature > dry, which is opposite to the trends observed for uniaxial compressive strength and elastic modulus. Higher moisture content enhances the plastic deformation ability of CPB, leading to an increase in strain at the same stress. However, as the loading rate increases, the influence of moisture content on peak strain diminishes. At higher loading rates, the failure process is mainly dominated by brittle fracture, and the effect of moisture is overshadowed by the accelerated loading rate.

### 3.3. Effect of loading rate and moisture content on the failure morphology of CPB

**Fig 9:**
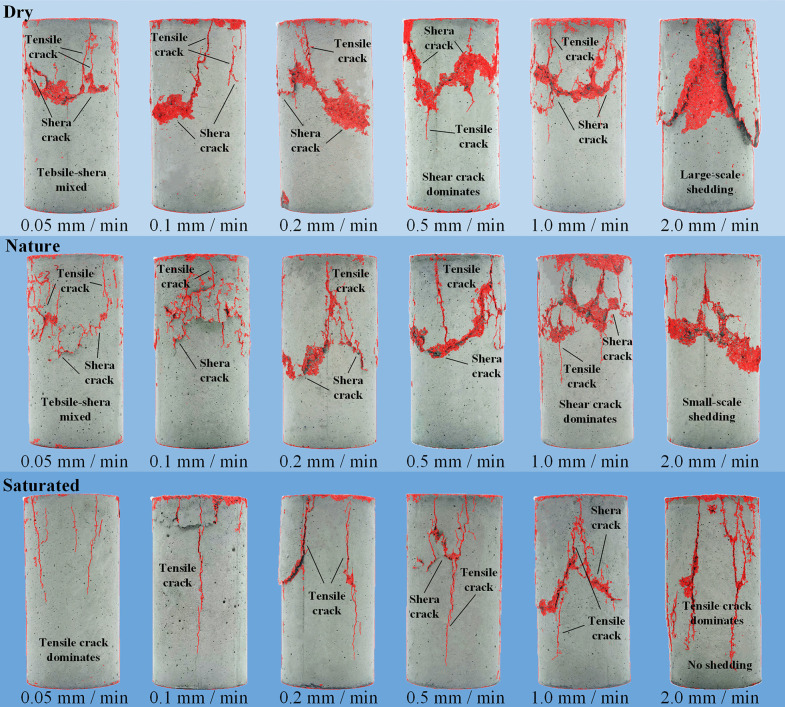
Failure characteristics of CPB under different conditions.

Fig 9 shows the failure morphology of CPB under different loading rates and moisture contents. Observation of failure cracks and regions under uniaxial compression shows that both loading rate and moisture content significantly affect the macroscopic failure morphology of CPB. The failure morphology mainly presents mixed tensile–shear failure, yet considerable differences appear as loading rate and moisture content change.

In the dry state, the failure morphology primarily exhibits mixed tensile–shear failure accompanied by regional spalling. As the loading rate increases, the failure mode transitions from mixed tensile–shear to mainly shear failure, with increasing regional spalling and pronounced brittle characteristics;

In the nature state, the failure morphology also exhibits mixed tensile–shear failure. With increasing loading rate, regional spalling gradually increases, but compared with the dry state it is markedly reduced and the brittle characteristics are weakened. The crack propagation path is more complex, and the failure regions are more dispersed, showing certain ductile characteristics;

In the saturated state, the failure morphology primarily exhibits mixed tensile–shear failure. As the loading rate increases, the morphology remains predominantly tensile failure, with only minor shear features and no surface spalling.

### 3.4. Failure mechanisms of CPB under moisture–loading rate coupling

Across the dry, nature, and saturated conditions, the UCS, elastic modulus, and peak strain exhibit a rise-then-fall trend with increasing loading rate, suggesting a common transition region within the tested range. In the present dataset, this region is centered near 1.0 mm/min [31–34]. Moisture condition and loading rate both significantly affect the mechanical response of CPB and jointly govern its deformation and failure behavior before and after the transition region.

**Fig 10:**
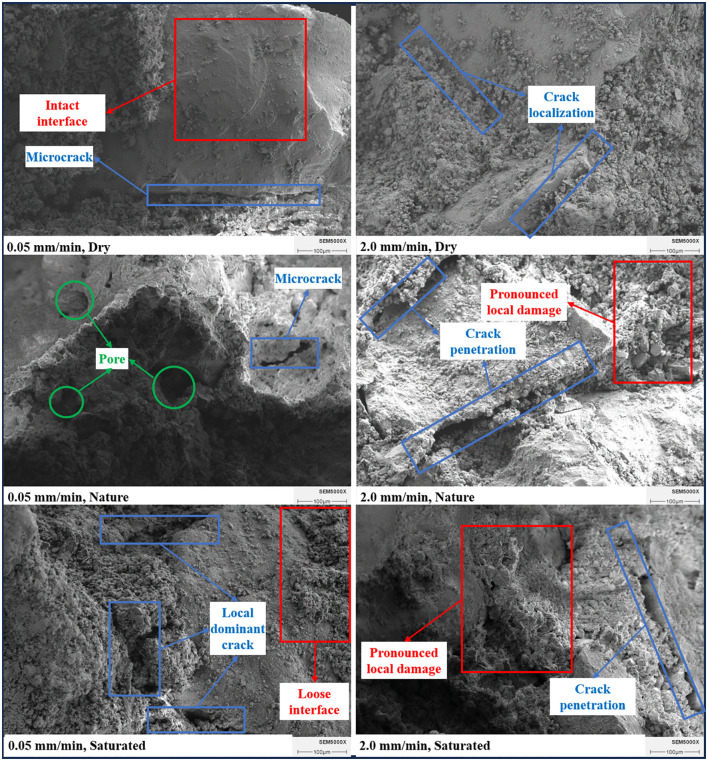
SEM images of CPB fracture surfaces under different moisture conditions and loading rates.

Moisture condition significantly affects the mechanical response of CPB, and the corresponding microstructural differences can be observed on both sides of the common transition region, although they are more evident before the transition region. As shown in Fig 7, increasing moisture content prolongs both the OA compaction stage and the BC yielding stage, resulting in greater pre-peak deformability. The saturated specimens exhibit more pronounced pre-peak deformation and nonlinear response. As shown in Fig 10, compared with the dry specimens, the saturated specimens display looser particle–matrix interfaces, more evident local debonding, and greater pore exposure, indicating weaker structural integrity and compactness. These micromorphological features are consistent with the macroscopic trends of reduced strength and enhanced deformation. Meanwhile, the failure mode gradually shifts from shear-dominated failure in the dry state to more tensile-dominated failure in the saturated state, indicating that moisture condition affects not only the load-bearing capacity of CPB, but also its crack propagation pattern and final failure morphology.

Loading rate is another key factor governing the mechanical response of CPB. As shown in Figs 5 and 7, before the common transition region, a lower loading rate allows more sufficient internal adjustment, thereby leading to gradual increases in strength and stiffness. Once the loading rate exceeds the transition region, damage evolves more abruptly, as reflected by reduced peak strength, lower peak strain, and enhanced brittleness. The SEM images in Fig 10 show that, under the same moisture condition, specimens tested at 0.5 mm/min exhibit more dispersed microcracks and relatively intact interfaces, whereas those tested at 2.0 mm/min show clearer interfacial separation, more localized crack propagation, and more pronounced local damage. These micromorphological observations are consistent with the macroscopic transition from relatively distributed deformation to more localized damage.

In mining practice, loading rate and moisture condition often vary simultaneously, and loading-rate regulation in underground backfill-supported environments should therefore be considered from a coupled perspective. The loading-rate selection and regulation should be adjusted according to the prevailing moisture condition rather than based on a single fixed criterion.

## 4. Discussions

### 4.1. Engineering criterion for moisture–rate coupling

Underground CPB is persistently subjected to the combined action of slow stress evolution (surrounding-rock convergence and mining-induced disturbance) and moisture variation (seepage, ponding during shutdown, and drainage-induced drying). The loading rate can be regarded as an effective proxy for disturbance intensity/frequency, whereas moisture content governs interfacial friction within the granular skeleton and the level of pore pressure. Their coupling directly controls the trade-off among bearing capacity (strength/stiffness), deformability (ductility/peak strain), and failure morphology, thereby shaping parameter selection and on-site safety margins.

To quantitatively characterize the effect of “moisture–rate” coupling on the mechanical response of CPB, this study adopts the nature moisture condition as the baseline and first defines a moisture effect coefficient [35]:


$$\begin{array}{c} \overset{\left(Q\right)}{\underset{w}{K}}\left(r\right)=\frac{Q_{w}\left(r\right)}{Q_{nat}\left(r\right)} \end{array}$$ (3)

Where *w* is the moisture state (dry, nature, saturated), *r* is the loading rate (mm/min), *Q* is the mechanical response, *Q*_*w*_(*r*) is the value of *Q* at moisture state *w* and rate *r*, and the *Q*_*nat*_(*r*) is the value of *Q* at the nature moisture state and the same rate *r*. *K* > 1 denotes enhancement relative to the nature state (higher strength/stiffness or greater ductility), *K* < 1 denotes deterioration.

Within a fixed moisture state, we introduce the rate-sensitivity coefficient [36–38]:


$$\begin{array}{c} \overset{\left(Q\right)}{\underset{w}{S}}\left(r_{i}\right)\approx \frac{ln\;Q_{w}\left(r_{i+1}\right)-ln\;Q_{w}\left(r_{i}\right)}{ln\;r_{i+1}-ln\;r_{i}} \end{array}$$ (4)

Where *r*_i_, *r*_i+1_ are adjacent loading rates, *S* is the local logarithmic slope that reflects the direction and magnitude of the response change with rate; *S* > 0 indicates an increase with rate, *S* < 0 a decrease, and the zero crossing marks the turning point of a “rise-then-fall” trajectory.

Based on this, we state a unified critical-rate criterion [39]:


$$\begin{array}{c} r_{c}=\text{arg}\underset{r}{\text{max}}\sigma_{c,nat}\left(r\right)\text{0.17em}or\text{0.17em}\;\overset{\left(\sigma_{c}\right)}{\underset{nat}{S}}\left(r_{c}\right)=0 \end{array}$$ (5)

Where *r*_*c*_ is the critical loading rate, *σ*_*c*,nat_(*r*) is the UCS under nature moisture; If *r*_*c*_ remains nearly identical across moisture states while the peak magnitude and the flanking slopes differ systematically, this indicates a “fixed critical point with moisture-modulated amplitude”.

To separate pure moisture effects from pure rate effects, we further define the rate–moisture coupling index [40,41]:


$$\begin{array}{c} \overset{\left(Q\right)}{\underset{cpl,w}{K}}\left(r|r_{ref}\right)=\frac{Q_{\left(w\right)}\left(r\right)/Q_{nat}\left(r\right)}{Q_{\left(w\right)}\left(r_{ref}\right)/Q_{nat}\left(r_{ref}\right)} \end{array}$$ (6)

Where *r*_*ref*_ is a reference rate, *K*_*cpl*_ ≈ 1 implies negligible modulation of the moisture effect by rate, values >1 denote amplification, and values <1 denote suppression.

To quantify the combined effects of strength, stiffness, and deformability on the mechanical performance of CPB under coupled loading-rate and moisture conditions, a unified coupled performance index (UCPI) was established. Since deterioration in any individual indicator may reduce the overall performance, the UCPI was formulated as a weighted geometric mean to enhance sensitivity to the weakest-link effect [42,43]:


$$\begin{array}{c} UCPI_{w}\left(r\right)={\left({\left[\overset{\left(\sigma_{c}\right)}{\underset{w}{K}}\left(r|r_{ref}\right)\right]}^{\alpha_{\text{1}}}{\left[\overset{\left(E\right)}{\underset{w}{K}}\left(r|r_{ref}\right)\right]}^{\alpha_{\text{2}}}{\left[\overset{\left(\varepsilon_{P}\right)}{\underset{w}{K}}\left(r|r_{ref}\right)\right]}^{\alpha_{\text{3}}}\right)}^{1/(\alpha_{\text{1}}+\alpha_{\text{2}}+\alpha_{\text{3}})} \end{array}$$ (7)

Where *α*_1_, *α*_2_, *α*_3_ ≥ 0 are weights reflecting engineering preference for strength, stiffness, and ductility; *UCPI*>1 indicates a net gain relative to the reference rate, and *UCPI*<1 indicates suppression.

For deployment, we define the safety loading window [44,45]:


$$\begin{array}{c} W_{w}=\left\{r\in \left[r_{min,}r_{max}\right]|\sigma_{c,w}\left(r\right)\geq \sigma_{thr},\varepsilon_{p,w}\left(r\right)\geq \varepsilon_{thr,}UCPI_{w}\left(r\right)\leq K_{max}\right\} \end{array}$$ (8)

Where [*r*_min_, *r*_max_] is the feasible rate domain under process constraints, *σ*_*thr*_ is the minimum load-bearing threshold (MPa), *ε*_*thr*_ is the minimum ductility threshold, and *K*_max_ is an upper bound set to suppress adverse coupling; this yields, for a given moisture scenario, the recommended rate interval that simultaneously satisfies bearing, ductility, and coupling constraints, directly aligning with field monitoring and parameter selection.

After min-max normalization of uniaxial compressive strength, elastic modulus, and peak strain, the indicator weights were determined using the CRITIC method, which considers both indicator variability and correlation conflict. The corresponding equations are given as follows:


$$\begin{array}{c} X_{n}=\frac{X-X_{min}}{X_{max}-X_{min}} \end{array}$$ (9)

$$\begin{array}{c} s_{j}=\sqrt{\frac{\text{1}}{n}\sum_{i=1}^{n}{\left(\overset{*}{\underset{ij}{x}}-\overset{*}{\underset{j}{\overset{―}{x}}}\right)}^{\text{2}}},C_{j}=s_{j}\sum_{k=1}^{m}\left(1-r_{jk}\right),w_{j}=\frac{C_{j}}{\overset{m}{\underset{j=1}{\sum }}C_{j}} \end{array}$$ (10)

Where 𝑋 is the raw data value, 𝑋_min_ and 𝑋_max_ are the minimum and maximum values of the indicator, respectively; *s*_*j*_ is the standard deviation of the *j*-th indicator, *r*_*jk*_ is the correlation coefficient between the *j*-th and k-th indicators, *C*_*j*_ is the amount of information contained in the *j*-th indicator, and *w*_*j*_ is the objective weight determined by the CRITIC method.

As shown in Tables 4–6 peak strain has the highest information content and therefore receives the greatest CRITIC weight, with the weights of UCS, elastic modulus, and peak strain being 0.23, 0.26, and 0.51, respectively. Relative to the nature condition, the dry condition generally enhances UCS and elastic modulus, whereas the saturated condition weakens them but promotes pre-peak deformability over the low-to-intermediate loading-rate range. Under all three moisture conditions, CPB exhibits a rise-then-fall response with increasing loading rate. For UCS, the enhancement under the dry condition peaks near 1.0 mm/min, whereas the weakening under the saturated condition is alleviated at intermediate rates but intensifies at higher rates. Consistently, the rate-sensitivity coefficient becomes negative within 1.0–2.0 mm/min for all moisture conditions, indicating the onset of deterioration, while the rate–moisture coupling index further confirms that loading rate modulates the effects of drying and saturation on CPB behavior. Overall, the effect of moisture condition on CPB is strongly loading-rate dependent, indicating that loading rate governs not only the absolute mechanical response of CPB but also the extent to which drying and saturation modify that response.

**Table 4:** Objective weights determined by the CRITIC method.

Indicator	Standard deviation	Conflict coefficient	Information content	Weight
UCS	0.2551	1.2635	0.3223	0.2333
Elastic Modulus	0.2195	1.6134	0.3542	0.2564
Peak strain	0.2733	2.5796	0.7051	0.5103

**Table 5:** Moisture effect coefficient *K* under different loading rates and moisture conditions.

Loading rate（mm/min）	*K* based on UCS			*K* based on Elastic Modulus			*K* based on Peak Strain		
	Dry 0%	Nature 18%	Saturated 32%	Dry 0%	Nature 18%	Saturated 32%	Dry 0%	Nature 18%	Saturated 32%
0.05	1.057	1.000	0.844	1.283	1.000	0.707	0.946	1.000	1.080
0.1	1.068	1.000	0.821	1.084	1.000	0.808	0.851	1.000	1.167
0.2	1.090	1.000	0.829	1.084	1.000	0.678	0.938	1.000	1.117
0.5	1.152	1.000	0.915	1.245	1.000	0.882	0.921	1.000	1.094
1.0	1.203	1.000	0.918	1.424	1.000	0.926	0.831	1.000	1.065
2.0	1.004	1.000	0.775	0.789	1.000	0.754	1.171	1.000	0.854

**Note:** *K* is referenced to the nature condition; *K* ＞ 1indicates enhancement, and *K* ＜ 1indicates weakening.

**Table 6:** Rate-sensitivity coefficient *S* and rate-moisture coupling index *K*_*cpl.*_

Interval or Loading rate（mm/min）	Rate-sensitivity coefficient S			Rate-moisture coupling index Kcpl		
	Dry 0%	Nature 18%	Saturated 32%	Dry 0%	Nature 18%	Saturated 32%
0.05-0.1	0.089	0.074	0.033	–	–	–
0.1-0.2	0.117	0.087	0.101	–	–	–
0.2-0.5	0.105	0.045	0.152	–	–	–
0.5-1.0	0.116	0.054	0.059	–	–	–
1.0-2.0	−0.373	−0.112	−0.357	–	–	–
0.05	–	–	–	1.000	1.000	1.000
0.1	–	–	–	1.011	1.000	0.973
0.2	–	–	–	1.032	1.000	0.982
0.5	–	–	–	1.090	1.000	1.084
1.0	–	–	–	1.138	1.000	1.088
2.0	–	–	–	0.950	1.000	0.918

Note: *S* was calculated between adjacent loading rates; *K*_*cpl*_ was calculated relative to 0.05 mm/min.

**Fig 11:**
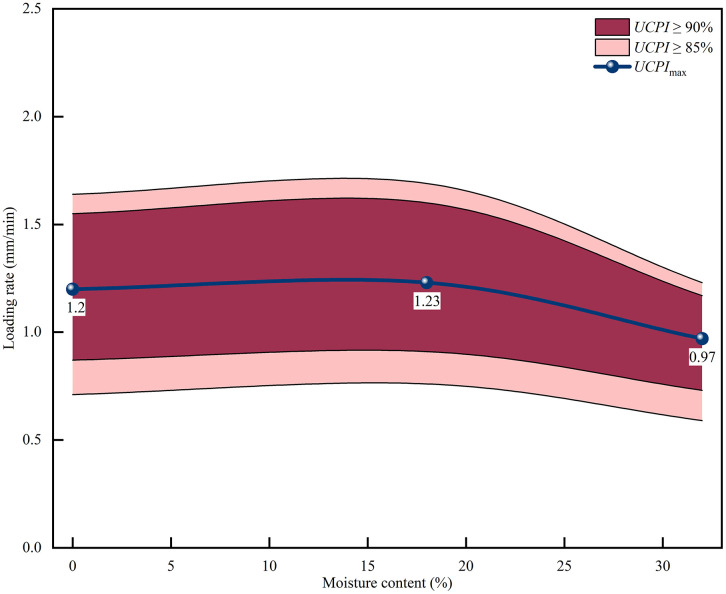
*UCPI*-Rate Curves and Recommended Operating Windows for Different Moisture Contents.

As shown in Table 7, the normalized *UCPI* under all three moisture conditions exhibits an overall rise-then-fall trend with increasing loading rate. At the discrete test points, the normalized *UCPI* reaches its maximum at 1.0 mm/min for all moisture conditions and remains relatively high over 0.5–2.0 mm/min, indicating that this interval represents a high-performance range within the tested loading-rate range. Quadratic fitting and extremum analysis of the *UCPI*–loading-rate relationship were further performed to determine the continuous optimal loading rates and corresponding operating windows, as summarized in Table 8. The fitted optimal loading rates are 1.20, 1.23, and 0.97 mm/min for the dry, nature, and saturated conditions, respectively. Although these values are not identical, they are all clustered around 1.0 mm/min, suggesting that 1.0 mm/min is more appropriately interpreted as a common transition region within the tested range rather than a strictly unique optimum applicable to all moisture conditions. Fig 11 further shows that the saturated condition exhibits a narrower and left-shifted preferred operating window, whereas the dry and nature conditions retain relatively wider windows, indicating that elevated moisture content suppresses the composite mechanical performance.

**Table 7:** Normalized *UCPI* values under different moisture conditions.

Loading rate（mm/min）	Normalized *UCPI* value (%)		
	Dry 0%	Nature 18%	Saturated 32%
0.05	83.39	83.27	76.72
0.1	83.20	91.34	89.99
0.2	92.74	96.46	89.07
0.5	97.07	97.86	97.81
1.0	100.00	100.00	100.00
2.0	94.38	96.92	79.91

Note: *UCPI* was normalized to its peak value (100%) for each moisture condition.

**Table 8:** Optimal loading rates and recommended operating windows based on *UCPI.*

Moisture condition (%)	Fitted optimal loading rate (mm/min)	Preferred operating window (UCPI ≥ 90%)	Safety lower-bound window (UCPI ≥ 85%)
Dry 0%	1.20	0.87-1.55	0.71-1.64
Nature 18%	1.23	0.91-1.60	0.76-1.69
Saturated 32%	0.97	0.73-1.17	0.59-1.23

For engineering application, loading-rate regulation should be adjusted according to the moisture-dependent UCPI window rather than a single fixed rate criterion, with saturated conditions generally requiring lower and narrower operating ranges.

Table 9 shows that, although the fitted optimal loading rates vary to some extent under different weighting schemes, they remain clustered around 1.0 mm/min for all three moisture conditions. This indicates that the present interpretation of a common transition region and moisture-dependent performance differentiation is reasonably robust.

**Table 9:** Sensitivity analysis of optimal loading rates under different weighting schemes.

Weighting scheme	Optimal loading rate for Dry 0% (mm/min)	Optimal loading rate for Nature 18% (mm/min)	Optimal loading rate for Saturated 32% (mm/min)
Equal weights (0.33/0.33/0.33)	1.07	1.27	1.03
CRITIC objective weights (0.23/0.26/0.51)	1.20	1.23	0.97
Engineering-oriented weights (0.50/0.30/0.20)	1.04	1.26	2.05
Strain-biased weights (0.20/0.20/0.60)	1.37	1.19	0.93

### 4.2 Nonlinearity metrics and engineering implications

The nonlinear index is an essential indicator for evaluating the degree of nonlinearity in the compressive behavior of cement-based backfill materials. This index quantifies the degree of nonlinearity in the stress-strain curve. The higher the nonlinear index, the stronger the nonlinearity of the CPB. It can, to some extent, reflect the influence of loading rate and moisture content on the nonlinearity of the CPB specimen [46,47]. The nonlinear index is defined as follows:


$$\begin{array}{c} N=\left|1-\frac{E_{40}}{E_{100}}\right| \end{array}$$ (11)

Where *N* is the nonlinear index of the CPB, *E*_40_ is the tangent modulus at 40% of the peak stress on the stress-strain curve, and *E*_100_ is the deformation modulus from the origin to the peak stress point.

**Fig 12:**
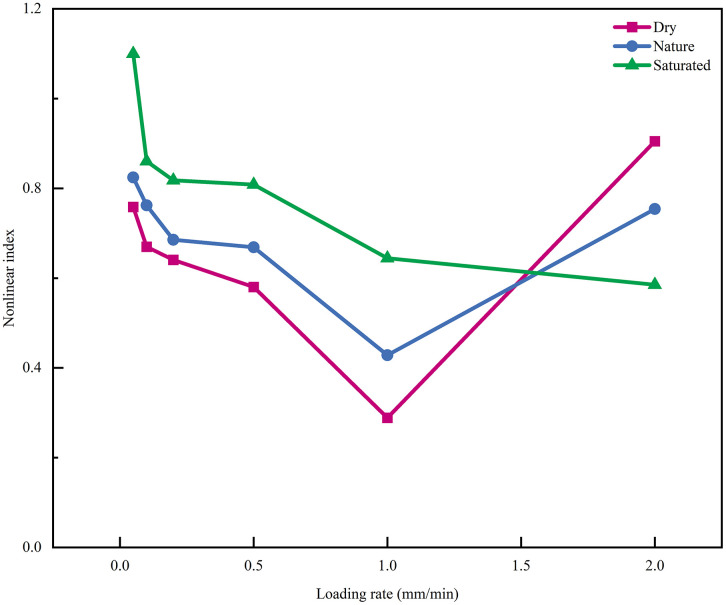
Nonlinear Index of CPB under Different Conditions.

The nonlinear index of CPB under different loading rates and moisture contents is shown in Fig 12. From the figure, it can be observed that before the transition region, the CPB with three different moisture contents exhibits the highest nonlinear index in the saturated state. This behavior may be associated with water weakens and lubricates the CPB, which may promote more distributed deformation prior to peak stress. In other words, after the elastic phase. the CPB may still undergo a more extended nonlinear deformation stage before peak stress, showing an increased nonlinearity and pre-peak deformability as the moisture content increases.

Overall, increasing loading rate suppresses strain nonlinearity, with the nonlinear index of CPB decreasing as the rate rises. At low rates, particles have time to rearrange and stresses to redistribute, so cracks develop cooperatively in a progressive manner and the stress–strain curve shows greater curvature. At high rates, the timescales of external loading and microstructural coordination are mismatched, contact forces concentrate, and cracks rapidly coalesce, which drives the response toward linearity. Beyond the transition region, the degree of nonlinearity follows a consistent order in which the dry state exceeds the nature state, and the nature state exceeds the saturated state. In high-moisture conditions, pore water pressure may weaken effective contact forces and may promote stress localization, causing earlier crack initiation and penetration and thus depressing nonlinearity; in the dry state, load transfer relies on particle bonding and friction, delaying crack penetration and preserving a higher level of nonlinearity. Related scholars have discussed the post-peak behavior of the backfill under various conditions, which to some extent is broadly consistent with the above-mentioned findings [48,49].

In comparison with peak strain, the nonlinear index better reflects the pre-peak curvature of the stress–strain response, but it should still be considered a supplementary rather than a definitive measure of ductility. A rigorous evaluation of ductility would still require post peak or energy-based indicators, such as post peak area comparison, toughness integral, or energy absorption.

From an engineering perspective, the nonlinear index may provide supplementary information for evaluating deformation compatibility before peak loading. However, backfill design and parameter selection should not rely on this indicator alone, and future work should incorporate post-peak and energy-based metrics to enable a more comprehensive assessment of deformability and safety performance.

## 5. Conclusions

The loading rate and moisture content significantly affect the mechanical properties, failure mode, and nonlinear characteristics of CPB. This study conducted UCS tests on CPB samples under different loading rates and moisture contents, and the following conclusions were drawn:

(1) Under all tested moisture conditions, the UCS of CPB exhibits a rise-then-fall trend with increasing loading rate and follows a second-order polynomial relation. The mechanical response indicates a common transition region around 1.0 mm/min. Before this transition region, increasing moisture content generally reduces the strength and stiffness of CPB; beyond this region, the combined influence of loading rate and moisture condition becomes more pronounced.
(2) Loading rate and moisture condition jointly affect the failure morphology of CPB. Under dry conditions, CPB exhibits more brittle behavior, and the failure mode gradually shifts from mixed tensile–shear to shear-dominated failure with increasing loading rate. Under nature conditions, crack propagation is relatively more dispersed. Under saturated conditions, CPB exhibits higher peak strain and more tensile-dominated failure, with limited surface spalling.
(3) Based on UCPI analysis, the fitted optimal loading rates are 1.20 mm·min ⁻ ¹, 1.23 mm·min ⁻ ¹, and 0.97 mm·min ⁻ ¹ for the dry, nature, and saturated conditions, respectively. Although the fitted optima differ slightly among moisture conditions, they remain clustered around 1.0 mm·min ⁻ ¹, indicating a common transition region within the tested range. The UCPI-based operating windows provide a quantitative basis for selecting and adjusting loading rates under different moisture scenarios. In addition, the nonlinear index is jointly governed by loading rate and moisture condition, with the saturated state exhibiting the strongest pre-peak nonlinear response. This index may provide supplementary information for evaluating deformation compatibility.

## Supporting information

S1 File Minimal dataset underlying the results reported in this study. (XLSX)
